# Using built-in functions of Adobe Acrobat Pro DC to help the selection process in systematic reviews of randomised trials

**DOI:** 10.1186/s13643-016-0207-7

**Published:** 2016-02-18

**Authors:** Selin Nur, Clive E. Adams, David F. Brailsford

**Affiliations:** Information Systems in Healthcare Management, Department of Informatics, Ulm University of Applied Sciences/Neu-Ulm University of Applied Sciences, Ulm, Germany; Cochrane Schizophrenia Group, Institute of Mental Health,, University of Nottingham, Triumph Road, Nottingham, NG7 2TU UK; School of Computer Science, University of Nottingham, Nottingham, NG7 2TU UK

**Keywords:** Systematic reviews, Automation, Text mining, Portable Document Format (PDF)

## Abstract

This letter describes a simple way of using Adobe Acrobat Pro DC to help select and auto-extract data from Portable Document Format (PDFs) of randomised trials in order to assist swift early selection of trials for a systematic review.

## Background

Automated extraction of data from randomised trials of the effects of healthcare is attractive [1]. Systematic reviews contain tabulated data often extracted from source Portable Document Format (PDFs). It is rare that these tabulated data contain explicit source co-ordinates and are rarely shared. Without transparency, the systematic nature of the work is threatened. Without the potential to share, maintenance is needlessly repetitive. There is the potential gain of saving time of [expensive] researchers by extracting from documents with some common structure. However, automated extraction of all study data still requires development for maximal accuracy [2] and may be impossible. This leaves the current reviewers with a problem. Although the hope of ‘jam tomorrow’ is attractive, the reviewers have to deal with the ‘bread and butter’ of routine and manual extraction.

The process of data extraction for a review is, in reality, staged. Stage 1 screens database output (decision—acquire/not acquire full text), i.e. study selection based on title and abstract—involving the lowest level of extraction. Stage 2 involves full text, frequently in PDF—the decision being whether to include/exclude the study, i.e. more detailed study selection combined often with extraction of the non-numeric data justifying the decision. Thereafter, stage 3 commences with full-data extraction. Recognising that stages 1 and 3 may be beyond our basic computing skills, we decided to experiment with Acrobat 11 Pro to see if it can assist in stage 2, i.e. the stage by which study selection is undertaken and basic non-numerical data are extracted to support the selection decision. Other systems exist (Apache Gate, Dr Evidence) but are less ubiquitous than the Acrobat packages.

## Methods

We downloaded Adobe Acrobat Pro DC and piloted techniques on a subset of reports. The Cochrane Schizophrenia Group holds all reports of relevant randomised trials in either PDF—Formatted Text and Graphics (PDF-FTG) or PDF Image plus Hidden Text (PDF-IT) format [3]. We converted all PDF Image Only (PDF-I) files to PDF-IT using the built-in Optical Character Recognition (OCR) facilities in Acrobat, from version 7 onwards.

Using the Action Wizard function, we created a .*TXT* file holding ‘target words’ on which selection of a trial for a particular review is undertaken (stage 2). The length of the list of ‘target words’ should be short so as not to over-clutter the PDF with mark-up—thereby decreasing the value of the eventual highlight (Table 1).

**Table 1:** Example of the structure of the TXT file for one review

Target word	Target of the word
Random	Methods
Blind	
Schiz	Participants
Tropis	Interventions
Placeb	
Cognit	Outcomes

Words may be truncated as Acrobat highlights the whole word in which these letters occur

Adobe Acrobat Pro DC allows the batching of a series of commands into one. We used this to merge ‘Find’, ‘Highlight’ and ‘Create Comment Summary’ commands (in ‘Actions List’ within the ‘Action Wizard’ tool). (If they do not exist already in the Action Wizard, there is an option to download the required functions from the Web.) Once the PDF (PDF-IT or PDF-FTG but not PDF-I) is uploaded, the new action can be run.

## Results

Adobe Pro DC creates a *separate* PDF file in which the target words are highlighted and linked to their comment.

**Fig. 1:**
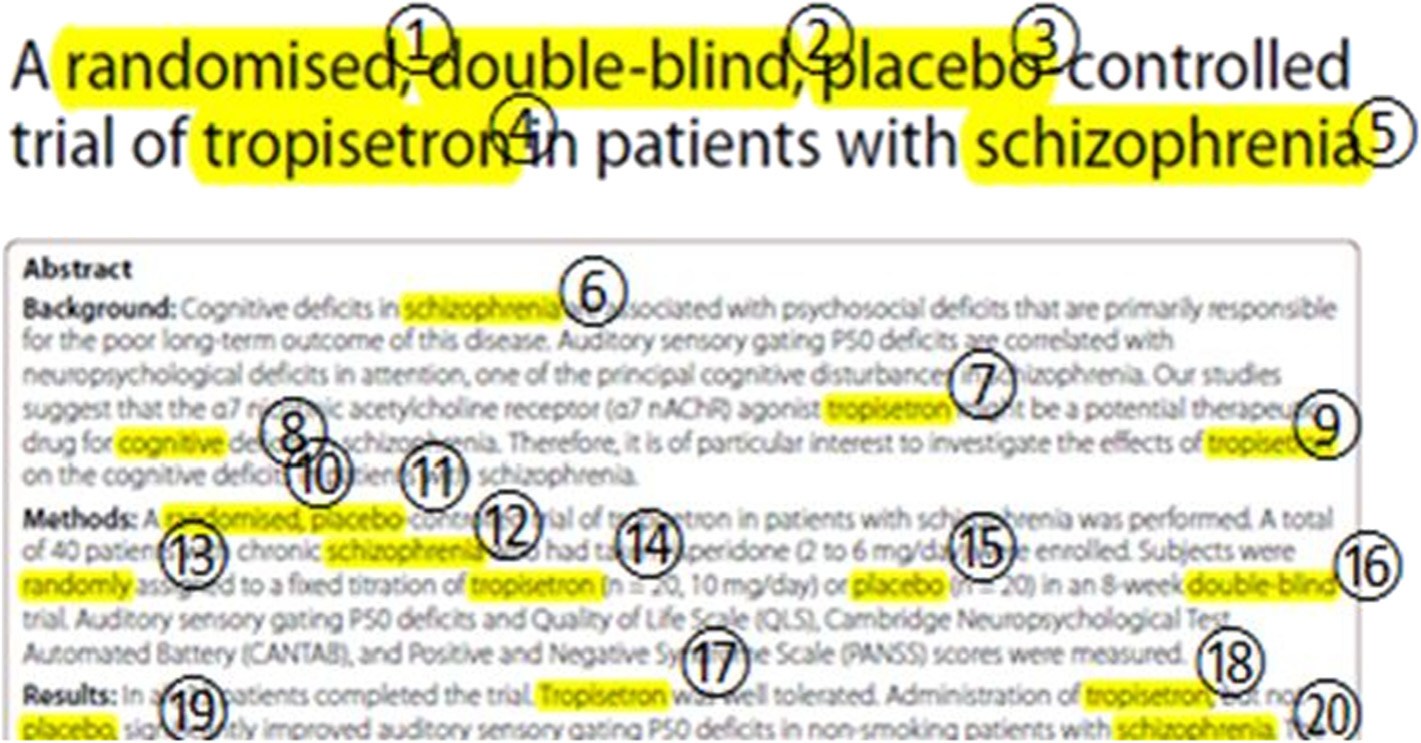
Example of annotated page from a paper [4]

The comment takes the form of a full-text word targeted as a result of the initial Acrobat text list (Acrobat highlights the complete word in which the target pattern of letters is found) and a numerical annotation (Fig. 1). The targeted word and the annotation also are listed after each of the original PDF’s text pages. Acrobat allows several options for creating a summary of the comments. One option links the target words by the use of lines drawn across the PDF. Each line contains the accurate coordinates of the target words, and it is possible to go beyond the simple selection of the word and extract that specific target word *and* coordinates into a table. Currently, this is too manual a process but it gives us a glimpse of the ‘Holy Grail’ of data extraction—where accurate, data extraction creates a sharable machine-readable table with source co-ordinates of each piece of information.

Finally, Acrobat has an option to create a comment summary in MS Excel. This tabulates target words with the page number—although, currently, not the exact co-ordinate where the word occurs. This can be edited to something like Table 2 in seconds.

**Table 2:** MS Excel table collated and transformed within MS Word

Page	PICO	Target word
1	Methods	Divided
1		Random
1		Random
2		Methods
3, 4, 5	Participants	Schizophrenia
3		Schizoaffective
1, 2, 3, 4, 5	Intervention	Chlorpromazine
2	Intervention	Reserpine
2	Results	Result
3, 4, 5		Results

## Summary

Part of the manual process within systematic reviews of healthcare by which data are identified and extracted for consideration can feasibly be replaced by using simple actions in Adobe Acrobat Pro DC. For a given review, the manual process can take considerable time. Batch processing in Acrobat Pro takes seconds, and the resulting extracted non-numerical data are traceable to source. Further work should compare full-text study selection, performed blinded and in parallel by two experienced reviewers, with disagreements resolved by a third reviewer who is blinded to which reviewer used the software.

## Abbreviations

OCR: Optical Character Recognition
PDF: Portable Document Format
